# LAMP in Neglected Tropical Diseases: A Focus on Parasites

**DOI:** 10.3390/diagnostics11030521

**Published:** 2021-03-15

**Authors:** Juan García-Bernalt Diego, Pedro Fernández-Soto, Antonio Muro

**Affiliations:** Infectious and Tropical Diseases Research Group (e-INTRO), Biomedical Research Institute of Salamanca-Research Centre for Tropical Diseases at the University of Salamanca (IBSAL-CIETUS), Faculty of Pharmacy, University of Salamanca, 37007 Salamanca, Spain; juanbernalt95@usal.es (J.G.-B.D.); ama@usal.es (A.M.)

**Keywords:** LAMP, neglected tropical diseases, parasites, point-of-care diagnostic

## Abstract

Neglected Tropical Diseases (NTDs), particularly those caused by parasites, remain a major Public Health problem in tropical and subtropical regions, with 10% of the world population being infected. Their management and control have been traditionally hampered, among other factors, by the difficulty to deploy rapid, specific, and affordable diagnostic tools in low resource settings. This is especially true for complex PCR-based methods. Isothermal nucleic acid amplification techniques, particularly loop-mediated isothermal amplification (LAMP), appeared in the early 21st century as an alternative to PCR, allowing for a much more affordable molecular diagnostic. Here, we present the status of LAMP assays development in parasite-caused NTDs. We address the progress made in different research applications of the technique: xenomonitoring, epidemiological studies, work in animal models and clinical application both for diagnosis and evaluation of treatment success. Finally, we try to shed a light on the improvements needed to achieve a true point-of-care test and the future perspectives in this field.

## 1. Neglected Tropical Diseases Caused by Parasites: The Diagnostic Limitation

In 2005, the “Neglected Tropical Diseases” (NTDs) concept was defined by researchers David H. Molyneux, Peter J. Hotez and Alan Fenwick. They grouped thirteen infectious diseases, caused by bacteria and parasites, that fitted a common definition: “A poverty-promoting and often stigmatizing condition occurring primarily in rural areas of low income countries” [1]. Since then, the World Health Organization (WHO) has updated that list to twenty conditions caused by bacteria, viruses, parasites, and snake envenoming affecting some of the world’s poorest communities, predominantly in Africa, Asia, and the Americas. Those living without adequate sanitation and in close contact with infectious vectors, domestic animals and livestock are worst affected (https://www.who.int/neglected_diseases/diseases/en/, accessed on 1 December 2020).

**Figure 1:**
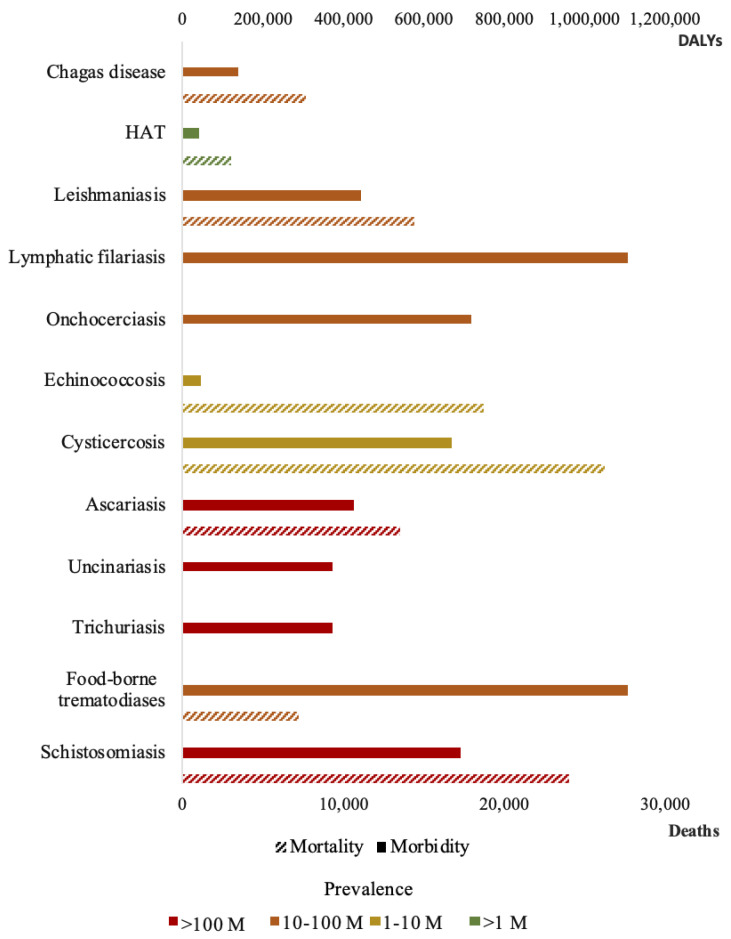
Estimated burden of parasite-caused Neglected Tropical Diseases (NTDs). Solid bars represent disability adjusted life years (DALYs) data, obtained from Kyu et al. [3]. Cross-lined bars represent mortality data, obtained from Global Health Estimates (GHE) 2016 [6]. Color of the bars represents prevalence values (referred as M, million people), obtained from Hotez et al. [7].

Of the 20 NTDs recognized by WHO, 12 are caused by parasites (parasite-caused NTDs): Chagas disease (American trypanosomiasis), Dracunculiasis (Guinea-worm disease), Echinococcosis, Foodborne trematode infections, Human African trypanosomiasis (sleeping sickness), Leishmaniasis, Lymphatic filariasis (Elephantiasis), Onchocerciasis (river blindness), Scabies and other ectoparasites, Schistosomiasis (Bilharzia), Soil-transmitted helminthiases, and Taeniasis and Cysticercosis (https://www.who.int/neglected_diseases/diseases/en/, accessed on 1 December 2020). It is likely that all of the world’s population living below the World Bank poverty line of US$1.90 per day are infected with one or more of these NTDs, corresponding to, at least, 10% of the global population [2]. Based on data provided by the 2017 Global Burden of Disease Study (GBD), Kyu et al. [3] calculated that over 17 million disability adjusted life years (DALYs) are caused by NTDs. This represents 4.7% of the total DALYs by any communicable, maternal, neonatal or nutritional disease [3]. Among the parasite-caused NTDs, the most prominent morbidities came from lymphatic filariasis, foodborne trematodiases, and schistosomiasis. On the other hand, the deadliest diseases according to the Global Health Estimates (GHE, 2016) by the WHO are schistosomiasis, cysticercosis, and echinococcosis, each causing over 20,000 deaths a year (https://www.who.int/healthinfo/global_burden_disease/en/, accessed on 25 November 2020). Global attention tends to focus on killer diseases, although NTDs disable and disfigure more than they kill [4]. A summary of the morbidity and mortality data for parasite-caused NTDs is shown in Figure 1. While all those numbers offer context to the current situation in tropical regions, it is important to emphasize the fluctuations that epidemiological data suffer. This is especially critical in tropical regions where data collection remains a very demanding task, and vast underestimations of both incidence and mortality have been reported in previous editions of the GBD [5].

In the new WHO road map for NTDs (2021–2030) a particular focus is directed to diagnosis monitoring and evaluation, access and logistics, and advocacy and founding to attain the sustainable development goals. The engagement of companies and foundations has been key to support advances in schistosomiasis, soil-transmitted helminthiases, or HAT diagnosis. However, the overall investment in diagnosis development has been very low, representing only 5% of research and development investment for NTDs, which as a hole, has decreased 10% in the last 10 years [8]. Although significant improvements have been made regarding chemotherapy, the same has not happened to the diagnostic tools to guide it. Classical NTDs diagnosis is hampered by the lack of a gold-standard and, in general, the lack of sensitivity and specificity. Accurate diagnostic tests are commercially available but are mostly laboratory-based, thus, not widely accessible in low-income countries [9]. PCR-based methods are expensive and infrastructure-demanding, therefore, not ideal for point-of-care (POC) tests. In this context, methods of isothermal nucleic acid amplification technology (INAAT) have emerged as a promising alternative. They can be performed with simple equipment, combining the sensitivity and specificity of molecular methods with a reduced response-time and cost. These methods have been recently reviewed by Zhao et al. [10]. Among them, LAMP (loop-mediated isothermal amplification) has become the preferred method, due to its sensitivity, specificity, rapidity, low cost and resistance to inhibitors [11,12] and represents 60% of all INAAT publications [13]. There are numerous well-established applications of LAMP technology in the diagnostic of bacterial, viral, fungal, and parasitic diseases in humans, animals, and plants [14,15]. Compared to PCR-based techniques, the simplicity of LAMP makes it suitable for field-testing in developing countries [16,17] and an ideal candidate to develop POC molecular diagnostic tools. In recent years, a great variety of approaches of the LAMP technology in a field-friendly display have been released, such as, lateral flow dipstick and lab-on-chip layouts [18], microfluidic-based methods [19], in combination with metallic nanoparticles [20], or coupled with smart phone-based technology [21].

## 2. Loop-Mediated Isothermal Amplification

**Figure 2:**
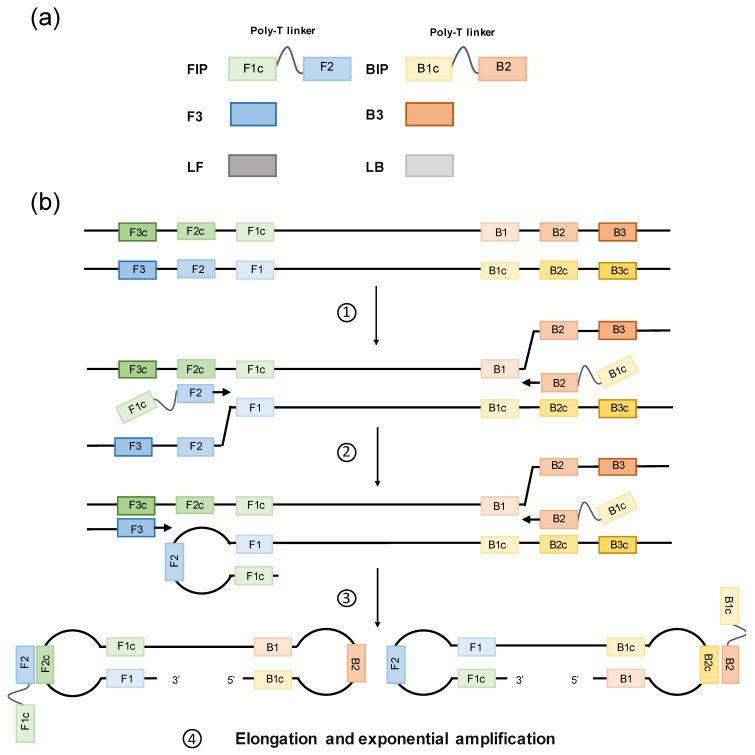
Loop-mediated isothermal amplification (LAMP): primer design and mechanism. (**a**) A typical set of LAMP primers is represented. LAMP reaction requires four primers, two inner primers (forward inner primer (FIP) and backward inner primer (BIP)) and two outer primers (F3 and B3). FIP and BIP each contains two sequences (usually linked by a poly-T linker) corresponding to the sense and antisense sequences of the target DNA. Additional loop primers (loop-forward (LF) and loop-backward (LB)), can be included, shortening the reaction time up to 30 minutes. (**b**) LAMP amplification process can be divided into two phases. At the first phase: 1. FIP hybridizes to the target DNA and *Bst* polymerase starts complementary strand synthesis. 2. The outer primer F3 starts strand displacement of the elongate FIP primer, releasing single stranded DNA (ssDNA). That ssDNA is used as template for the backward primers. The inner primer BIP hybridizes and starts strand synthesis at the ssDNA and then is displaced by the B3 primer. 3. Now, as the 3′ and 5′ ends are complementary to sequences further inwards, stem-loops DNA structures are formed and subsequently used as targets to start an exponential amplification second phase. 4. In the second phase, self-priming and the elongation of 3′ end induces displacement of the 5′ end and subsequently, the hairpin comes off and the newly synthesized strand folded. Further self-priming repetitions generate many amplicons with cauliflower-like structures. In addition, FIP and BIP primers now hybridize to the loop structures formed and initialize strand synthesis and subsequent displacement.

LAMP method was first introduced by Notomi et al. in 2000 [11] and was patented by Eiken Chemical Co., Ltd. (http://www.eiken.com.cn/, accessed on 1 December 2020). LAMP is based on auto-cycling strand displacement DNA synthesis performed under isothermal conditions (60–65 °C for 45–60 min) in the presence of a *Bst* polymerase [11]. In silico designed *Bst* mutants have been developed to improve processivity, fidelity, stability, and tolerance to amplification inhibitors, thus increasing robustness of the LAMP technique [22]. The LAMP reaction requires four primers (two inner and two outer primers), which specifically recognize six distinct sequences in target DNA, thus ensuing high specificity for amplification. The inner primers are called forward inner primer (FIP) and backward inner primer (BIP), and each contains two sequences (usually linked by a poly-T linker) corresponding to the sense and antisense sequences of the target DNA. The outer primers are called forward outer primer (F3) and backward outer primer (B3) (see Figure 2a). The amplification process can be divided into two phases. At the first phase, FIP hybridizes to the target DNA and *Bst* polymerase stars complementary strand synthesis. The F3 starts strand displacement of the elongate FIP primer, releasing single stranded DNA (ssDNA). That ssDNA is used as template for the backward primers. The BIP hybridizes and starts strand synthesis at the ssDNA and then is displaced by the B3 primer. Now, as the 3′ and 5′ ends are complementary to sequences further inwards, stem-loops DNA structures are formed and subsequently used as targets to start an exponential amplification second phase (see Figure 2b). In the second phase, self-priming and the elongation of 3′ end induces displacement of the 5′ end and subsequently, the hairpin comes off and the newly synthesized strand folded. Further self-priming repetitions generate many amplicons with cauliflower-like structures. In addition, FIP and BIP primers now hybridize to the loop structures formed and initialize strand synthesis and subsequent displacement. This method operates on the fundamental principle of the production of a large quantity of DNA amplification products with a mutually complementary sequence and an alternating, repeated structure [11,23]. Nagamine et al. [24] introduced loop primers (LF, loop-forward; LB, loop-backward), thus shortening the reaction time by approximately 30 min. Consequently, a six-primer design can be used in LAMP reactions (two inner, two outer and two loop primers), targeting in up to eight different sequences, compared to only two in typically PCR-based methods. For ease of explanation see animations at: http://loopamp.eiken.co.jp/e/lamp/anim.html; www.neb.com, accessed on 1 December 2020.

## 3. LAMP Development in Parasite-Caused NTDs

**Figure 3:**
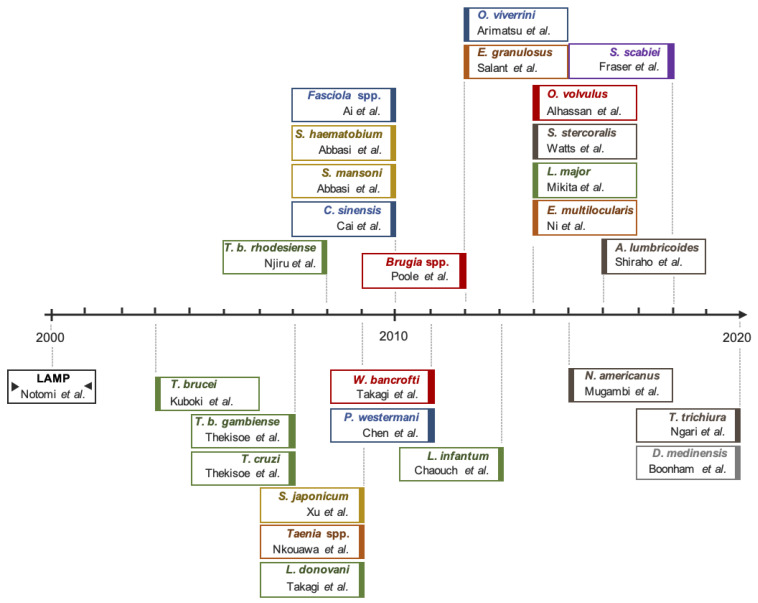
Timeline of first LAMP tests development for parasite-caused NTDs. Color code; orange, Cestodiases: *Taenia* spp., *Echinococcus granulosus* (*E. granulosus*) *Echinococcus multilocularis* (*E. multilocularis*); blue, Food-borne trematodiases: *Fasciola* spp., *Clonorchis sinensis* (*C. sinensis*) *Paragonimus westermani* (*P. westermani*), *Opistorchis viverrini* (*O. viverrini*); brown, Soil-transmitted helminthiasis: *Strongyloides stercoralis* (*S. stercoralis*), *Necator americanus* (*N. americanus*), *Ascaris lumbricoides* (*A. lumbricoides*), *Trichuris trichiura* (*T. trichiura*); green, Protozoa: *Trypanosoma brucei* (*T. brucei*), *Trypanosoma brucei gambiense* (*T. b. gambiense*), *Trypanosoma brucei rhodesiense* (*T. b. rhodesiense*), *Trypanosoma cruzi* (*T. cruzi*), *Leishmania infantum* (*L. infantum*) and *Leishmania major* (*L. major*); grey, Dracunculiasis: *Dracunculus medinensis* (*D. medinensis*); red, Lymphatic filariasis: *Brugia* spp., *Wuchereria bancrofti* (*W. bancrofti*) and *Onchocerca volvulus* (*O. volvulus*); violet, Scabies: *Sarcoptes scabei* (*S. scabei*); yellow, Schistosomiasis: *Schistosoma mansoni* (*S. mansoni*), *Schistosoma haematobium* (*S. haematobium*) and *S. japonicum* (*S. japonicum*). References in the figure: Notomi et al. [11], Kuboki et al. [25], Thekisoe et al. [26], Njiru et al. [27], Xu et al. [28], Nkouawa et al. [29], Ai et al. [30], Abbasi et al. [31], Cai et al. [32], Takagi et al. [33], Takagi et al. [34], Chen et al. [35], Arimatsu et al. [36], Salant et al. [37], Poole et al. [38], Chaouch et al. [39], Alhassan et al. [40], Watts et al. [41], Mikita et al. [42], Ni et al. [43], Mugambi et al. [44], Shiraho et al. [45], Fraser et al. [46], Ngari et al. [47], Boonham et al. [48].

The potential of LAMP as a possible POC diagnostic test for NTDs was clear from the publication of the technique in the year 2000 and, in 2003, the first LAMP assay for human African trypanosomes DNA detection was published [25], representing the start of a new diagnostic approach for NTDs. Since then, many researchers have reported LAMP assays for parasite-caused-NTDs as an alternative molecular tool to PCR-based methods. However, to date, only a few of the methods developed have been tested in real field conditions. A timeline of first LAMP assays described for each parasite-caused NTD is show in Figure 3.

Over the years, LAMP assays development and evaluation have followed similar steps for most parasite-caused NTDs: (1) target selection, primer design, set-up, and optimization with purified parasite genomic DNA (gDNA) and synthetic DNA; (2) feasibility of application in different specimens; (3) clinical application using patients’ samples; and (4) improvements towards a true POC test. Other relevant studies needed for test development in particular parasite-caused NTDs are the assessment of the efficacy testing intermediate hosts or vectors as xenomonitoring tool, and evaluation in experimental animal models. Those complementary studies allow the assessment of important variables like, inclusivity (recognition of different strains or genotypes of the same species) and exclusivity (discrimination between species) values, the evaluation of treatment success, early-stage of infection diagnosis, infection dynamics, or species-specific identification.

### 3.1. Genomic Target Selection and LAMP Optimization

**Figure 4:**
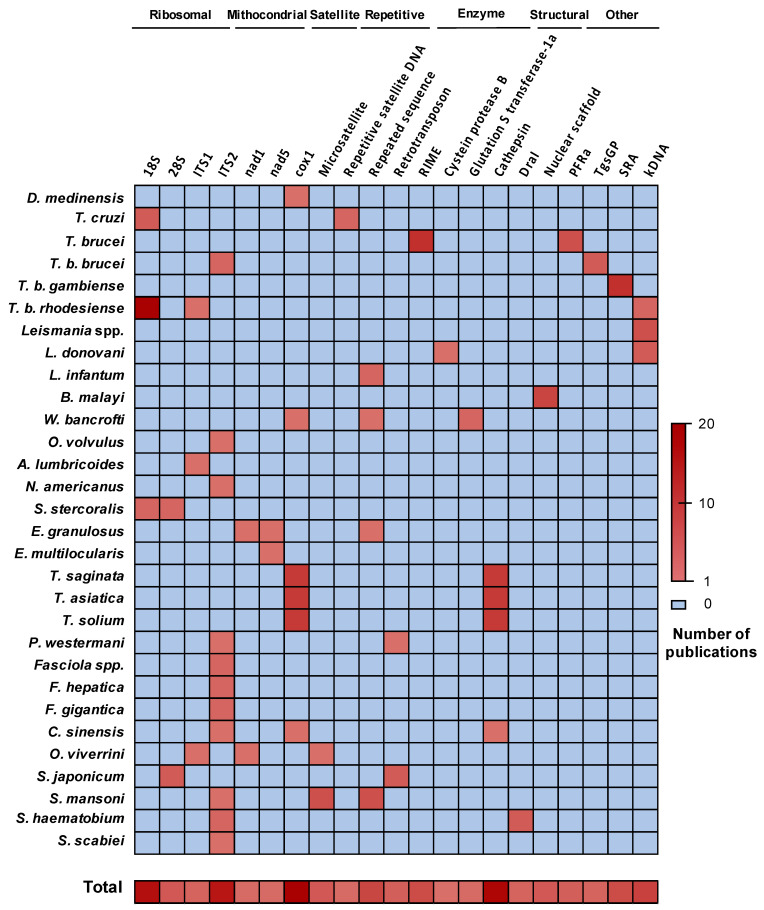
Heat map of the target sequences used for LAMP in parasite-caused NTDs. Sequence abbreviations, from left to right: Microsat (Microsatellite), Rep sat (Repetitive Satellite), Rep Seq (Repetitive sequence), Retrotrans (Retrotransposon), RIME (Repetitive insertion mobile element), CpB (Cystein protease B), GST1a (Glutation-S-Transferase 1a), Nucl scaffold (Nuclear scaffold); PFRa (Paraflagellar Rod) TgsGP (*T. b. gambiense* specific gene,) SRA (serum-resistance associated gene) kDNA (kinetoplast DNA). Data obtained from all the publications used in this review.

Similar to other molecular-based diagnostic methods, selection of genomic targets focusses on highly specific and highly repeated sequences to obtain both high specificity and sensitivity. Some targets are commonly used for nearly all species, particularly nuclear ribosomal sequences, including the internal transcribed spacers 1 and 2 (ITS1 and ITS2), the intergenic spacer (IGS) and the 18S and 28S ribosomal DNA (18S rRNA and 28S rRNA); and mitochondrial sequences, such as NADH dehydrogenase subunits 1 and 5 (nad1 and nad5) or cytochrome c oxidase subunit 1 (cox1). The principal exception is concerning Leishmania species where the main target used is the highly repeated kinetoplast DNA (kDNA). Other frequently targeted sequences are satellite sequences, retrotransposons and genes coding for structural proteins or enzymes (Figure 4). LAMP primer design is more complex than for PCR. There are various systems for LAMP primer design available but the most popular is Primer Explorer, an online free software (https://primerexplorer.jp/e/).

A large number of LAMP studies working with parasite gDNA have consistently shown that LAMP reaches at least similar sensitivity values to those obtained with PCR-based methods. Remarkably, some studies have shown up to 100 to 1000 times more sensitivity for LAMP than PCR, as in the detection of *Paragonimus westermani* using the ITS2 DNA region [35] and *Clonorchis sinensis* targeting the cathepsin B3 gene [32], that reach a limit of detection as low as 10 ag/µL. Nevertheless, most LAMP assays reach sensitivities ranging from 1 to 100 fg/µL.

### 3.2. LAMP in Molecular Xenomonitoring

A number of studies have shown the usefulness of LAMP as a disease and transmission surveillance tool, especially valuable in low-prevalence areas, where other conventional techniques often lack sensitivity and accuracy. To date, LAMP has been successfully used for the detection of parasites in their insect vectors, including, triatomines (Chagas disease), tsetse flies (HAT), sandflies (leishmaniasis), mosquitoes (LF), black flies (onchocerciasis) and also snail intermediate hosts for schistosomiasis Particularly valuable results have been reported in transmission assessment surveys (TAS) for detection of *Wuchereria bancrofti* DNA in Anopheles and Culex mosquitoes collected in regions of Guinea [49] and Nigeria [50]. In those areas, the transmission of LF-causing parasites is suggested to be unsustainable due to the decline, or absence, of circulating filarial antigen (CFA) in the population. However, *W. bancrofti* DNA was detected by LAMP in mosquitoes, which had tested negative by microscopy, PCR, or both [49,50]. These results lead to the recommendation that filarial infection prevalence in the human and mosquito populations should be re-assessed periodically. Further valuable are a number of studies of the LAMP method for detecting schistosomes-infected intermediate host snails. In experimental infections of snails, LAMP could detect parasite DNA during the prepatent phase of infection (as soon as one day after miracidial exposure) in both individual snails and pooled samples [51]. LAMP has also been evaluated for the detection of *Schistosoma* species in large-scale screening of pooled field-collected snails for analyzing the transmission of schistosomiasis, especially in low-transmission areas. The results of these studies agree that LAMP is a rapid, sensitive, and a cost-effective tool to screen large numbers of snail samples compared with other PCR-based methods. The usefulness of LAMP to identify foci of transmission in order to build risk maps of schistosomiasis is also apparent in these publications and could be a contributing factor in control campaigns [52,53,54,55,56].

### 3.3. LAMP in Experimental Infections

Within the main limitations of both classical microscopy and serology diagnosis are the inability to detect acute infections and the irregular performance through the course of the parasite infections. In vivo studies in animal models have repeatedly shown the value of LAMP as an early-detection diagnostic tool in comparison to PCR-based methods. Regarding HAT, LAMP could detect *T. b. gambiense* in mice blood samples as soon as two days post-infection (dpi) and throughout the course of the infection, whereas PCR became positive at day 6 dpi and yielded an irregular infection detection [25]. In *T. b. gambiense*-infected monkeys monitoring during 180 dpi, LAMP and PCR were compared using serum, cerebrospinal fluid (CSF), saliva, and urine samples that were collected on a weekly basis, with a significantly higher efficiency of LAMP versus PCR in serum (100% at 7 dpi), saliva (100%; 21–77 dpi), and urine (80%; 28–91 dpi) [57]. In echinococcosis, when monitoring experimentally infected dogs, LAMP performed in faeces became positive at 22 dpi (vs. 26 dpi using PCR, 25 dpi using copro-ELISA, and 69 dpi using microscopy) for *E. granulosus* [58], and at 12 dpi (vs. 17 dpi using PCR and 44 dpi using microscopy) for *E. multilocularis* [43]. These results are very promising for echinococcosis, for which early detection and treatment can prevent hydatid cysts development. The increased sensitivity achieved using LAMP in comparison to PCR was also reported in the analysis of blood samples of dogs experimentally infected with *P. westermani* metacercariae, allowing three weeks earlier detection by LAMP than PCR (2 weeks p.i. vs. 5 weeks p.i.) [59]. In experimental schistosome-infection animal models, LAMP has been evaluated to detect *S. mansoni* DNA in serum, plasma [60], urine [61], and in stool samples [62,63] from infected mice, as well as DNA of *S. japonicum* in stool, serum [28,64], and blood samples [65] from infected rabbits. In all those studies, schistosome-derived DNA was detected in the acute phase of the infection, before the microscopic detection of parasite eggs in faeces [28,60,62] and even before other diagnostic methods, both immunological [62,64] and molecular [65]. This proves the high sensitivity of LAMP as a tool for monitoring active infections in many different biological specimens and a potential method for early diagnosis of human schistosomiasis.

### 3.4. LAMP in Clinical Studies

To date, most human clinical studies testing LAMP in parasite-caused NTDs are very limited and only a handful present a relatively large sample size. A selection of the most representative based on their sample size, results, and novelty is summarized in Table 1. It is imperative to carry on large-scale studies to further validate the LAMP technique. Nevertheless, a few studies are worth highlight. An example is the specific detection of *T. b. rhodesiense* using DNA eluted from FTA (Flinders Technology Associates) cards spotted with blood from HAT patients collected in Tanzania [66]. Clinical samples were tested by LAMP targeting RIME [27] and SRA [67] regions (see Figure 4) and by a *T. b. gambiense*-specific PCR. The high level of concordance (98.4%) and agreement (kappa value, 0.85) obtained between RIME-LAMP and SRA-LAMP demonstrated the possibility that either test could be used to reliably diagnose *T. b. rhodesiense*-HAT, needing five times less reaction time than PCR [66]. Another study for HAT diagnosis using RIME-LAMP was conducted in Uganda on blood samples from *T. b. gambiense* HAT patients [68]. RIME-LAMP was compared with other isothermal amplification test, the nucleic acid sequence-based amplification (NASBA) combined with oligo-chromatography (OC) [69]. NASBA-OC showed a significantly higher sensitivity than RIME-LAMP for *T. b. gambiense*, but LAMP needed less equipment and time compared to NASBA-OC.

Several studies using different *Leishmania*-LAMP approaches have widely evaluated the clinical utility of different human samples and different DNA extraction methods for cutaneous leishmaniasis (CL), visceral leishmaniasis (VL), and Post-kala-azar dermal leishmaniasis (PKDL) diagnosis in different endemic areas [70,71,72,73,74,75]. All the *Leishmania*-LAMP assays developed to date have been recently exhaustively reviewed and discussed by Nzelu et al. [76] and Silva et al. [77]. In general, bone marrow, and specially whole blood samples, achieve the higher sensitivity and specificity values, satisfying WHO standard rates [78].

With regard to soil-transmitted helminthiasis, the largest clinical study has been performed to detect *Necator americanus* in human stool samples comparing Kato-Katz (KK) with LAMP [44]. Comparison of both techniques showed an overall 97% clinical sensitivity for the LAMP assay.

In relation to taeniasis, a LAMP to detect *Taenia solium* was carried out in blood samples from patients suffering from neurocysticercosis (NCC) and results were compared in different clinical situations [79]. Conventional LAMP was adapted into a real-time assay showing higher sensitivity in patients with extra-parenchymal brain cysts (86.7%) than those with intra-parenchymal brain cysts (71.8%), all with high specificity (90.2%). The number of cysts did not significantly affect sensitivity. In addition, a higher sensitivity compared to enzyme-linked immunoelectrotransfer blot (EITB) when performed in patients with single cysts and patients with calcified cysts was recorded, thus showing a high value of the technique in any clinical situation.

Among foodborne trematodiases, LAMP-based assays have been used principally in clinical surveys of clonorchiasis and opistorchiasis. Rahman et al. [80] developed a LAMP for *Clonorchis sinensis* detection which was tested on human stool samples with confirmed infection both by two KK smears and one real-time PCR. The sensitivity and specificity of the LAMP relative to the combined result of KK and real-time PCR resulted 97.1% and 100%, respectively. The only two false-negative results obtained by LAMP had only 12 eggs per gram of faeces (EPG) by KK. This low number of eggs, combined with their heterogeneous distribution in stool, might have resulted in a lack of eggs in the sample portion used for DNA extraction. This slightly lower sensitivity would be tolerable as low-burden infections would rarely result in cholangiocarcinoma if untreated. In exchange, LAMP assay saves a minimum of 2 h to diagnosis and greatly reduce infrastructure required [80]. Additionally, it would also allow clinicians to reduce double-checks needed for an accurate diagnosis, thereby improving control programs and treatment. Concerning opistorchiasis, an *Opisthorchis viverrini*-LAMP was tested using stool samples microscopically selected from schoolchildren in Khon Kaen Province, Thailand. A diagnostic sensitivity of 100% with a proved analytical sensitivity of 1 pg/μL was obtained with LAMP. However, a poor clinical specificity of 61.5% was obtained, suggesting missed eggs by microscopy in light infections or cross-reactions with other organisms. This remains unclear as specificity was not evaluated in the development of this LAMP [36]. Those results showed a clear limitation in the gold standard diagnostic tool (light microscopy) used for comparison as, the differences in sensitivity between techniques lead to a very low specificity value (61.5%), that may not be an accurate representation of the diagnostic value of LAMP in the case of *O. viverrini* diagnosis.

Finally, several studies have evaluated the clinical application of LAMP in diagnosis of human schistosomiasis. LAMP has been applied to detect *Schistosoma haematobium* in field conditions using both purified DNA and heat-treated urine samples in comparison with microscopy in human urine samples collected in an endemic area in Cubal, Angola [81]. The overall prevalence detected by LAMP was significantly higher than microscopy when testing both purified DNA (73.8% vs. 50.6%) and crude urine samples (63.4% vs. 50.6%). The reproducibility of LAMP tests in a well-equipped laboratory decreased especially in crude urine samples, probably because of the inappropriate samples storage over time [81]. Another study evaluated a LAMP to detect *S. haematobium* DNA in urine samples collected from suspected patients for urogenital schistosomiasis attending outpatient clinic in Imbaba Cairo, Egypt. LAMP resulted in a 100% sensitivity and 63.16% specificity when compared with conventional urine filtration followed by microscopy for egg detection [82]. Regarding *S. mansoni* DNA detection in clinical stool samples, a first survey using LAMP was conducted in a low-transmission area in Umbuzeiro, Brazil [55]. Using KK as the reference test, LAMP resulted in an overall sensitivity of 92.86% and 80.11% specificity.

### 3.5. LAMP in Post-Therapy Monitoring

To date, very few studies have evaluated LAMP for monitoring the effectiveness of chemotherapy in parasite-caused NTDs. Stands out the work performed in *Schistosoma japonicum* infections in experimentally infected rabbits [28,64,65] and one in patients [64]. In the study conducted by Xu et al. [28], detection of *S. japonicum* DNA in rabbit sera infected with a high dose of 500 parasite cercariae became negative at 12-week post-infection after praziquantel administration. The results obtained by Wang et al. [65] in detection of *S. japonicum* DNA in experimentally infected rabbits with 200 cercariae following artesunate or praziquantel treatment demonstrated the higher sensitivity of LAMP compared to PCR for evaluation. *S. japonicum* DNA in rabbit sera remained detectable by PCR up 12 and 8 week post-treatment with artesunate and praziquantel, respectively, whereas DNA remained detectable by LAMP up 20 weeks in 50% and 66% of rabbit sera treated with artesunate and praziquantel, respectively. Finally, Xu et al. [64] evaluated the utility of LAMP assay for detection of light infections in experimentally infected rabbits and evaluation of chemotherapy efficacy both in animals and in patients. In this study, rabbits were infected using low infection doses (30 cercariae) and subsequently treated with praziquantel. LAMP could detect *S. japonicum* DNA in sera from infected rabbits as soon as the 3rd dpi and became negative at 10-week post-therapy, thus indicating the utility of LAMP in early diagnosis of light infection schistosomiasis, and in monitoring the effectiveness of treatment. In this same study, LAMP was also evaluated as a tool to assess the response to treatment in 47 patients’ sera infected with *S. japonicum* after treatment with praziquantel at 3, 6, and 9 months post-treatment. The parasite DNA in serum was not detected in 31.9%, 61.7%, and 83% at 3, 6 and 9 months post-therapy. IHA and ELISA only reached at nine months a conversion rate of 31.9% and 25.5%, respectively.

These studies indicate that the LAMP technique has potential for monitoring the effectiveness of schistosomiasis treatment. Nevertheless, further studies are needed with other parasite-caused NTDs to determine the usefulness of LAMP in assessing the efficacy of treatment and as a diagnostic tool after preventive chemotherapy campaigns.

**Table 1:** Summary of relevant studies of LAMP in parasite-caused NTDs.

Disease	Application ^1^				Specimen ^2,3^	Clinical Studies			Key Points
	VE	AM	HS	PT		n ^4^	Sensitivity	Specificity	
Dracunculiasis	✓	✓	✓	✕	N/A [48]	N/A	N/A	N/A	Test applied in adult worms recovered from humans, not in human specimens.
Chagas	✓	✓	✓	✕	Blood [83,84,85]	27 [83] 33 [84] 46 [85]	100% 73.9% 93%	100% 100% 100%	Accurate diagnosis in one test, regardless the clinical situation of the patient.
HAT	✓	✓	✓	✕	Blood [25,66,68,86] Buffy coat [87] CSF [87] Bone marrow [87] Sera [57] Saliva [57] Urine [57]	128 [66] 355 [86] 181 [68]	95.3–93.8% 87.3–93% 76.9%	N/T 92.8–96.4% 100%	Non-invasive samples such us saliva and urine useful substitutes of highly invasive CSF or bone marrow. Highly sensitive technique, fitting for the last stages of HAT control and elimination.
Leishmaniasis					Blood [70,71,72,73,74] Buffy coat [71] Saliva [75] Bone marrow [72] Skin [70,71,72]				One test can diagnose all presentations of leishmaniasis, in blood for VL and skin biopsies for CL or PKDL. However, invasive samples are still needed. Saliva might be a good alternative, but further studies are required.
VL	✓	✓	✓	✕		186 [73]	97.6–100%	99.1%	
						55 [72]	96.4%	98.5%	
						30 [70]	83%	100%	
						50 [71]	92.3%	100%	
						267 [74]	98.3%	96.6%	
CL	✓	✓	✓	✕		43 [70]	98%	100%	
						105 [71]	95%	86%	
PKDL	✓	✓	✓	✕		62 [72]	96.2%	98.5%	
Lymphatic filariasis	✓	✕	✓	✕	Blood [34]	N/A	N/A	N/A	Valuable for molecular xenomonitoring in low-prevalence areas and epidemiological control post-MDA ^5^.
Onchocerciasis	✓	✕	✓	✕	Skin [88,89]	70 [88] 146 [89]	65.7% 88.2%	N/T 99.2%	
Trichuriasis	✕	✓	✓	✕	Stool [47,90] Urine [90]	137 [47]	77%	88%	Urine might be a viable alternative to stool in epidemiological studies, but further evidence is needed. *Ancylostoma duodenale* does not have a specific LAMP designed yet.
Ascariasis	✕	✕	✓	✕	Stool [45]	40 [45]	96.3%	61.5%	
Uncinariasis	✕	✕	✓	✕	Stool [44]	106 [44]	97%	100%	
Strongyloidiasis	✕	✓	✓	✕	Stool [41,91,92] Serum [91] Broncho alveolar [91] Urine [92,93]	28 [41] 396 [91]	96.4% 77.4%	N/T 100%	
Echinococcosis	✕	✓	✓	✕	Stool [37,43,58] Hydatid cysts [94]	N/A	N/A	N/A	Good enough performance to avoid resource-demanding imaging techniques. Promising results in early infection detection, key in these diseases prognosis.
Taeniasis	✕	✕	✓	✕	Stool [95,96] Blood [79]	43 [95] 100 [79]	86% 74%	100% 90.2%	
Paragonimiasis	✓	✓	✓	✕	Blood [59] Sputum [35] Pleural fluid [35]	N/A	N/A	N/A	Larger studies with human clinical samples are required. Highly variable analytical sensitivity and specificity results.
Fascioliasis	✕	✓	✓	✕	Stool [97]	N/A	N/A	N/A	
Clonorchiasis	✓	✕	✓	✕	Stool [80]	120 [80]	97.1%	100%	
Opistorchiasis	✓	✕	✓	✕	Stool [36]	50 [36]	100%	61.5%	
Schistosomiasis					Plasma [60] Serum [28,60,64] Urine [61,81,98,99] Stool [28,55,62,63] Blood [65]	50 [28] 110 [64] 94 [98] 172 [81] 162 [55] 86 [99] 383 [63]	96.7% 95.5% 100% 86.2% 92.9% 100% 97%	100% 100% 86.7% N/T 80.1 100% 100%	Consistently shows similar or better performance than the other available diagnostic tools. Sufficient evidence in large clinical studies to start its implementation in public health of endemic and non-endemic regions
*S. japonicum*	✓	✓	✓	✓					
				
*S. haematobium*	✓	✓	✓	✕					
				
*S. mansoni*	✓	✓	✓	✕					
Scabies	✕	✓	✕	✕	Skin [46]	N/A	N/A	N/A	-

^1^ VE: vectors; AM: animal models; HS: human studies; PT: post-treatment studies. ✓ indicates that there are studies performed in this category; ✕ indicates there are no studies performed in this category. ^2^ In this category the parasite and the intermediate host are excluded. ^3^ Detection methods used were: SYBR Green I [28,35,36,37,44,45,47,55,57,60,61,62,63,64,66,68,72,74,80,81,83,87,90,92,93,97,98,99], electrophoresis [25,28,34,35,37,41,44,45,47,55,58,61,62,63,65,75,79,80,81,83,87,90,92,93,94,95,96,97,98,99], real-time detection [36,46,48,57,59,60,79,84,88,89,91,94], turbidity [34,57,71,80,88,96], calcein [84,85], hydroxynaphtol blue [36,88,94], fluorescence detection reagent (Eiken Chemical Co., Ltd.) [70,71,73,86], malachite green [75], neutral red [88], SYTO-82 [41], and lateral-flow dipstick [59]. ^4^ n: sample size. ^5^ MDA: massive drug administration. N/A: not applicable; N/T: not tested.

## 4. LAMP as Point-of-Care Test

**Figure 5:**
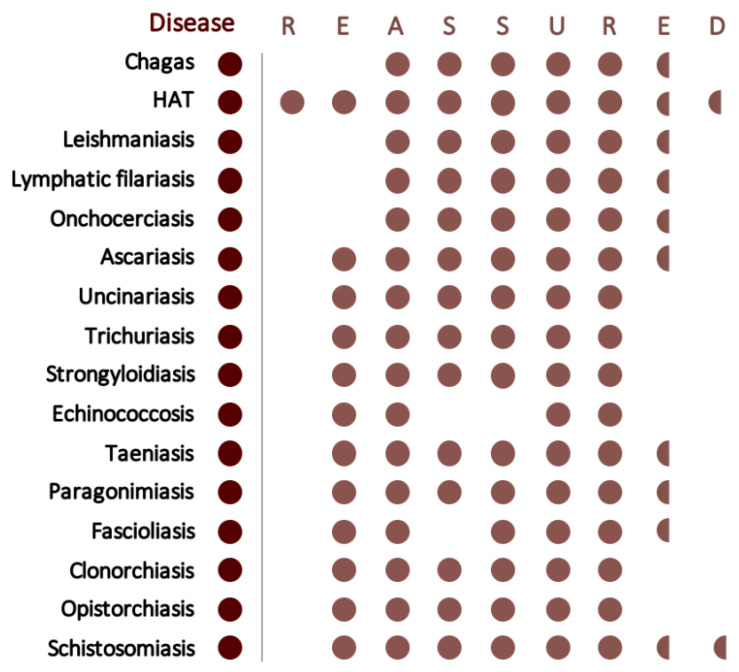
REASSURED criteria fulfillment of designed LAMP methods for the different parasite-caused NTDs. Red dots indicate that a criterion is fulfilled for the corresponding disease. Absence of dot means that criterion is not yet accomplished. Half-dots signify that steps have been taken to achieve those features; however, they are not yet met. An assay is considered sensitive with 75% or more clinical sensitivity. An assay is considered specific if no cross-reaction take place with other human infecting parasites. Dracunculiasis and scabies are excluded of this figure due to lack of sufficient information.

All parasite-caused NTDs, except dracunculiasis, have at least one available treatment and, access to those drugs, has significantly improved in recent years. However, accurate patient identification is still a major limitation in NTDs management and control, and dramatically contributes to the sustained burden they present worldwide [100]. Likewise, overtreatment or mistreatment, also consequence of a poor diagnostic capacity [101], lead to drug wasting and disease resurgence, respectively. Moreover, over-sustained treatment or under-dosage, which could be avoided using accurate diagnostic tools, could lead to drug resistance. Still, this is not due the lack of new diagnostic tools, rather the inability of those methods to reach low-resource settings. Particularly, molecular tools have not replaced classical methods, although consistently showing better results at the laboratory, being often more sensitive so needed as later stages of control. The challenge of affordable and simple molecular diagnostics development is not new, in 2002 was identified as the most important challenge of biotechnology contributing to improve developing countries health [102]. Since then, numerous techniques have been on the spotlight in parasite infections detection: real-time PCR (2002); LAMP (2003); multiplex ligation-dependent probe amplification (MLPA) (2005); high-resolution melt curve analysis (HRM) (2009); or digital PCR (dPCR) (2016). Despite of all this, none are actually routinely used in field settings [103]. In 2006, the acronym ASSURED (Affordable, Sensitive, Specific, User-friendly, Rapid and Robust, Equipment-free, Deliverable) was proposed by the WHO Sexually Transmitted Diseases Diagnostics Initiative (SDI) as a set of criteria that any diagnostic method must achieve to be considered as a POC test in low resource settings [104]. This term has been recently updated (so-called REASSURED), including: Real-time connectivity and Ease of specimen collection and Environmental friendliness [105] (Figure 5).

Unfortunately, frequently in tests development, achieving one of these features means trading-off another. When focusing on NAAT, concurrent accuracy, accessibility and affordability are almost never met. Higher accuracy usually leads to a lower accessibility and reduced affordability and vice versa. Thus, NAAT frequently present high accuracy, maybe valid for National Health Care Systems, but poor accessibility and affordability for communitarian and primary health care level [105]. Although LAMP has now become 20 years old [11], recent technological advances, might turn it into an ideal solution to those limitations, notwithstanding the necessary improvements still to be made for its deployment in resource-constrained settings.

### 4.1. Real-Time Connectivity

Smartphone technology accessibility is increasing exponentially worldwide, with over 60% adoption in 2017, and 70% expected in 2020. Even in Sub-Saharan Africa, where it was close to 40% in 2017, and 60% is expected in 2020 [106]. Smartphone-based diagnostic tests may provide useful applications for NTDs diagnostics in remote areas and greatly facilitate epidemiological surveys. A handheld digital microfluidic device for LAMP (so-called LampPort) with a fully Bluetooth control with a tablet has been recently presented as a proof of concept using *Trypanosoma brucei* as a model [107]. The on-chip detection sensitivity reached 40 DNA copies and endpoint naked-eye visualization was tested adding SYBR Green I. The system greatly reduced post-amplification contaminations but did not completely remove them, so further integration of sample preparation on-chip is needed [107]. Other smartphone-based LAMP systems have been also evaluated for other tropical diseases, including Zika, Chikungunya, and Dengue viruses [108] and malaria [109]. All these recent developments show a promising future, nonetheless, they should be carefully validated in clinical settings and compared to current diagnostic standards before implementation.

### 4.2. Ease of Specimen Collection

Two simultaneous requirements need to be met, a non-invasive specimen collection and little-to-non processing of the specimen pre-diagnosis. LAMP has proved to be as sensitive as real-time PCR without any prior nucleic acid purification in a great variety of body fluids (i.e., plasma, blood, urine, saliva, or semen) [110]. This feature has allowed to substitute invasive-specimen collection, such serum for *T. b. gambiense* detection, for non-invasive-specimen collection, such as urine or saliva, without compromising sensitivity of the test [57]. It has also allowed to use alternative specimens, such as urine samples, for molecular diagnosis of intestinal parasites, instead of stool samples, which are harder to handle and store [72,96]. Urine samples have already been successfully evaluated in LAMP assays for schistosomiasis [80,96,97] and strongyloidiasis [93] human diagnosis. Not only the specimen collection has improved, but also the sample processing has been reduced. This is of critical importance since nucleic acid purification is regarded as the primary bottleneck preventing adoption of NAAT outside the well-equipped laboratory [111]. Several approaches combining a simple DNA processing with LAMP have been applied for several parasites-caused NTDs without any need for costly laboratory instrumentation and skilled personnel. One example is the rapid-heat LAMPellet method (RHE-LAMP) for *Schistosoma haematobium* detection in clinical urine samples using only a 15 min 95 °C heat lysis step [98]. For *Leishmania infantum* detection, a simple boil-spin protocol with a rapid centrifugation and incubation at 90 °C has been used to process heparinized blood samples [112]. More sophisticated strategies have focused on incorporating the nucleic acid extraction step into a microfluidic chip, reducing handling and potential contaminations, as is the case for *Schistosoma mansoni* detection [60]. In all, the combination of rapid nucleic acid extraction protocols and LAMP assays have been presented for the successful detection of *T. brucei* [66,113], *Leishmania* spp. [42,75,112], *Onchocerca volvulus* [40], *Taenia* spp. [114], *S. mansoni* [51,55,99,115], and *S. haematobium* [81,98].

### 4.3. Affordable

No benchmarks are settled on what is considered an affordable diagnostic test; however, $0.5–1 has been accepted for HIV and malaria while up to $10 for tuberculosis [105]. For parasites-caused NTDs, being vastly diseases of the poor, research should target the cheaper end of the spectrum. LAMP technology markedly reduces costs of molecular diagnostic compared to PCR-based methods. Equipment is reduced from an expensive thermocycler to a simple heating block, water-bath, or non-instrument nucleic acid amplification (NINA) heating devices [116]. As stated above, in LAMP assays, nucleic acid extraction can be by-passed thus reducing an important additional cost. Moreover, results detection, can be naked-eye visualized with Mg^2+^ dependent dyes, such as malachite green [117] or hydroxynaphtol blue [118], much cheaper than DNA-binding dyes or molecular probes. Estimates have been made for lymphatic filariasis diagnosis, whose LAMP assay for *W. bancrofti* detection, costs approximately $0.82 (PCR ≈ $2.20) [34]. For schistosomiasis diagnosis, the differences in price are even more significant. While LAMP only costs $0.71–2 per sample, PCR goes up to $6.4–7.7 and even, classical diagnostic techniques, such as, ELISA ($1.5) or KK ($2.00–2.67) are more expensive [119]. However, a bias can be attributed to this estimation as DNA purification is not considered when calculating LAMP pricing.

### 4.4. User-Friendliness

Two complementary solutions should be mentioned here: colorimetric detection of LAMP results and ready-to-use reaction formats. The colorimetric evaluation allows for untrained personnel to easily interpret the results. Regarding the latter, novel stabilized master mixes for LAMP reactions avoid cold chain maintenance and allow untrained personnel to easily perform the diagnosis. Currently, these “LoopAmp kits” are scarce, but their improvement is an ongoing task in the case of kinetoplastid parasites, including *T. cruzi* [84], *T. brucei* [86], and *Leishmania* spp. [73,112]. In a recent work, our group [120] presented a novel protocol for long-term preservation of LAMP master mixes for *S. mansoni* detection through a simple 30 min one-step protocol based on the use of threhalose as cryoprotectant to produce functional ready-to-use reaction mixes. Another dry-LAMP approaches for schistosomiasis based on a different cryoprotectants (i.e., sucrose) have also been reported [54].

### 4.5. Rapid and Robust

Since LAMP tests can be performed isothermally for 45–60 min using a wide variety of biological specimens, greater robustness and shorter run times are achieved compared to conventional PCR. The robustness of LAMP reactions has also been enhanced with the improvements in speed, sensitivity, inhibitors tolerance, or stability to enable room temperature set-up of the *Bst* DNA polymerases family (http://www.neb-online.de/wp-content/uploads/2015/04/NEB_isothermal_amp.pdf, accessed on 10 January 2021). Moreover, these novel engineered polymerases are also suitable for ready-to-use formats that enables long-term storage at ambient temperature [121]. Additionally, real-time LAMP assays are significantly faster than PCR, showing results as soon as 16 min for *T. cruzi* [83] or 18 min for *Leishmania* spp. [112] in comparison with 2–4 h employed by conventional PCR.

### 4.6. Equipment Free

LAMP is not completely equipment-free, but it greatly reduces it. If DNA extraction is avoided, centrifuges can be circumvented all together. Moreover, the use of dry-LAMP protocols for ready-to-use tests allow to maintain all reagents at room temperature until the reaction is performed, thus greatly reducing additional equipment needed in field settings. The two most common strategies to avoid any equipment is the use of microfluidic chips and lateral flow dipstick (LFD). Among the first group, the work of Wan et al. [122] in *T. brucei* for HAT diagnosis stands out. They developed a chip based on low-Tm molecular beacons DNA probes that allows a 10x reduction in reagent consumption, with a LAMP reaction time of 40 min and a sensitivity of 10 copies. Unfortunately, clinical studies for most of these methodologies are still lacking. A LFD, classically used in serological tests, can be combined with LAMP technology. Once again, an example is the combination of RIME-LAMP for the detection of *T. brucei* with a LFD. The LFD-RIME-LAMP is based on specific labeling F1c and B1c primers with a fluorescein isothiocyanate (FITC) and a biotinalyted molecular probe. Using LFD-RIME-LAMP in clinical samples, *T. brucei* was detected in both bone marrow and CSF [123]. LAMP has also been combined with a LFD for the detection of *P. westermani* DNA [59]. Other approaches have been tested under field settings as equipment-free solutions for LAMP assays, including non-instrumented nucleic acid amplification (NINA) devices for the detection of filarial parasites DNA [124] or the use of a simple kettle for *Taenia* spp. DNA detection [114].

### 4.7. Deliverable to End-Users

Despite all the recent advances above-mentioned, no significant changes in current diagnostic protocols for parasites-caused NTDs have included LAMP yet. The technique has been available since 2000 [11] and has not yet broken the barriers of true POC testing. In fact, neither have any of the other NAAT. It is worth highlighting that in the Report of the first meeting of the WHO Diagnostic Technical Advisory Group for Neglected Tropical Diseases, in 2019, LAMP was a recommended current diagnostic technique for HAT and schistosomiasis, and the need for POC nucleic acid amplification diagnostic methods was acknowledge for echinococcosis, foodborne trematodiases, and taeniasis/cysticercosis [125]. This might have an effect in the near future in the deployment of LAMP and other molecular assays to the field.

Currently, LAMP presents a number of limitations that need to be acknowledge: non-applicable for cloning, primer design is subject to more constrains than other NAAT, high risk of carry-over contamination, and multiplexing approaches for multiple pathogen detection are highly complex and poorly still developed [126]. Furthermore, the lack of an internal control to rule out extraction failures and evaluate the presence or absence of inhibitors in the sample is a crucial limitation. This is a must in RT-qPCR commercial kits; however, most LAMP kits, whether home-made or commercial, do not include them. The reason behind it is probably the primer design complexity and the lack of a standardized technology to multiplexing LAMP assays.

There are other limitations that affect the deployment of new diagnostic assays that are not directly related with the technique itself. As discussed above, the limited investing in diagnostic development, only 5% of the NTDs research founding, that has also decreased overall (10% over the last 10 years) [8], significantly hamper any kind diagnostic improvement. Additionally, in the absence of massive infrastructure or health care facilities development, NTDs control campaings require integrated approaches, that are often complex and chaotic, and thus attract less funding and political actor investment than a new vaccine or a theoretically perfect diagnosis. Local programs should be designed and performed by locals and, while program managers often based their strategies in the WHO or other international entities, there is sometimes need for tailor-made solutions. In the case of diagnostics, cost-effectiveness of a particular assay can only be addressed at a local level [127].

Specifically addressing LAMP, the multiplexing drawback is very concerning, since co-infections are frequent in endemic regions and often times obscure clinical diagnosis. On the bright side, some multiplex-LAMP (mLAMP) approaches are beginning to appear for detection of parasite-caused NTDs. An example is combining mLAMP with dot enzyme-linked immunosorbent assay (dot-ELISA) for discrimination of the three human *Taenia* species by labelling species-specific FIP primers with fluorescein isothiocyanate (FITC), digoxigenin (DIG), and tetramethylrhodamine (TAMRA), and BIP primers labelled with biotin [128]. Another interesting multiplexed approach is a two-stage isothermal amplification method in a microfluidic format that consist of a dubbed rapid amplification (RAMP) first stage follow by a second-stage LAMP assay. This assay has been designed in a 16-plex, 2-stage RAMP assay to concurrent detection in 40 min up to 16 different targets of DNA and RNA from different pathogens, including helminths such as *S. mansoni*, *S. hematobium*, *S. japonicum*, *Brugia malayi*, and *Strongyloides stercoralis*. This multiplexed assay could provide healthcare personnel in endemic areas with a molecular tool to detect multiple pathogens in a single sample without a need to send the sample to a reference laboratory [115].

Thus, there is a clear need to share knowledge within the diagnostics field in the developing world to facilitate the development and deployment of the latest molecular tools, that can be extremely valuable for improving global health [101].

## 5. Conclusions

Parasite-caused NTDs are the most predominant and still representing a major Public Health concern in many developing countries, with the highest rates of disease burden, particularly lymphatic filariasis and foodborne trematodiases. The need for novel fast, affordable, specific, sensitive, robust, and easy to use diagnostic tools is only increasing to support the efforts towards control and elimination. Theoretically, INAAT fulfills all these needs, but to date only LAMP has been used in all parasite-caused NTDs. For NTDs caused by protozoa, LAMP has been particularly promoted in recent years. “Loopamp amplification prototype kits” in a ready-to-use format are available for Chagas disease, leishmaniasis, and HAT. In this regard, an optimistic future is upon us. In general, studies performed on clinical evaluation of LAMP for parasite-caused NTDs due to helminths show highly variable sensitivity and specificity, according to the parasite species and type of samples analyzed. Particularly, for intestinal helminths infections diagnosis and urogenital schistosomiasis, is very difficult to determine the sensitivity and specificity of LAMP assays due to the lack of a true “gold-standard” against which to compare, considering the low sensitivity of stool-based microscopic methods or urine filtration methods, routinely used in field surveys. Taking into account the potential effectiveness of the LAMP assays in helminths-derived DNA detection in urine, as well as its easier handling, processing and storage in low-resource settings, the use of patients’ urine samples would be a good alternative approach for helminths molecular detection. Until now, all of the studies about LAMP for parasite-caused NTD diagnosis agree that the clinical application of LAMP technology should only be considered as a pilot test. We expect that the new supportive technology (such as LFD, microchips, lab-on-chips, portable fluorometers, or smartphone apps) will help to meet the proposed REASSURED criteria, allowing LAMP to reach those who need it most. Additionally, sustained and targeted funding and political support are needed to validate and implement the technique. Overall, the current merits of LAMP technology outweigh its disadvantages.
